# Treatment of Intertrochanteric Femur Fractures with Hip Arthroplasty in Older Patients: A Narrative Review of Indications and Outcomes

**DOI:** 10.3390/medicina57080763

**Published:** 2021-07-27

**Authors:** Tiago Martinho, Karl Stoffel

**Affiliations:** 1 Department of Orthopaedic Sports Medicine, Technical University of Munich, Ismaninger Street 22, 81675 Munich, Germany; tiago.martinho@mri.tum.de; 2 Division of Orthopedics and Trauma Surgery, Geneva University Hospitals, Rue Gabrielle-Perret-Gentil 4, 1205 Geneva, Switzerland; 3 Department of Orthopaedics and Traumatology, University of Basel, Petersgraben 4, 4031 Basel, Switzerland

**Keywords:** intertrochanteric fractures, trochanteric fractures, femoral fractures, internal fracture fixation, hemiarthroplasty, total hip arthroplasty, total hip replacement

## Abstract

Intertrochanteric femur fractures are common in older patients and often have a significant impact on disability. The treatment aims to achieve a rapid return to the prior functional level with a low rate of complications and mortality. Surgical management by internal fixation is the mainstay of treatment for most of these fractures. Even when treated with intramedullary nails, the overall complication rates are high, especially for unstable or highly comminuted fractures or in the presence of poor bone quality. Hip arthroplasty is an alternative in older patients with intertrochanteric femur fractures at high risk of fixation failure or with concomitant intraarticular pathologies. Especially patients whose condition precludes prolonged bedrest and who are at risk of significant deterioration if their locomotor function cannot be restored rapidly are likely to benefit from hip arthroplasty. The choice of the surgical technique mainly depends on the surgeon’s preferences and the fracture characteristics. Bipolar hemiarthroplasty is the most common type of prosthesis used with primary or revision femoral stems. Compared with intramedullary nails, hip arthroplasty has a better early functional outcome and lower rates of surgical complications as well as reoperations. However, the functional outcome and the mortality rate in the longer term tend to favor intramedullary nails, even though the results are inconsistent, and a statistically significant difference cannot always be obtained. Currently, there are no guidelines that define the role of hip arthroplasty in the treatment of intertrochanteric femur fractures in older patients. The literature only offers an overview of the possibilities of the usage of hip arthroplasty, but methodological limitations are common, and evidence levels are low. Further studies are needed to identify the intertrochanteric fractures that are at high risk of internal fixation failure, the characteristics that determine which patients may benefit most from hip arthroplasty, and the optimal surgical technique.

## 1. Introduction

Intertrochanteric femur fractures (IFF) are a major public health problem because of their frequency and the associated complications in older patients [1]. These fractures are often a reason for a reduced quality of life, loss of mobility, increased dependence, and mortality in the year following the injury regardless of the type of treatment [2].

In this already fragile population, the aim of the treatment is to achieve a rapid return to the prior functional level with a low rate of complications and mortality [3]. For these reasons, surgical management is the mainstay of treatment for the vast majority of IFFs. Of the various surgical options, internal fixation (IF) is often the preferred treatment modality [4]. For this purpose, there is a wide variety of extra- or intramedullary devices available on the market.

However, IF does not always achieve the desired treatment goals. Especially in IFFs with unstable fracture patterns (AO/OTA type 31.A2 and A3 or Evans type III, IV, and V), a high degree of comminution, and the presence of low bone quality, it is difficult to obtain an acceptable fracture reduction and a sufficiently stable fixation. This in turn affects the return to full weight-bearing and leads to a significant increase in the rate of complications. The development and improvement of intramedullary nails (IM), which are currently the devices of choice for these fractures, has led to better outcomes; however, they could not solve all problems [5].

Hip arthroplasty (HA) has therefore been adopted as an alternative to IF since the 1970s. Its theoretical advantages are the possibility of full weight-bearing immediately after surgery and the low rate of complications [6,7].

## 2. Indications

HA is recognized as a viable treatment option for IFFs with a high risk of IF failure. Fracture instability patterns, such as posteromedial cortex comminution, thin lateral wall thickness, subtrochanteric extension, and reverse obliquity but also severe comminution and osteoporosis, make fracture reduction more difficult and thus bear the risk of implant mispositioning, which ultimately increases the risk of implant failure [8,9]. Of note, the majority of IFFs can be successfully treated by IF even in the presence of these characteristics. Indeed, there is no preoperative diagnostic tool to unequivocally identify fractures at high risk of IF failure or those at risk for insufficiently stable fixation, which would not permit an early return to full weight-bearing. In this respect, surgeon experience and treatment preferences play an important role in the choice of the surgical technique [10].

**Figure 1:**
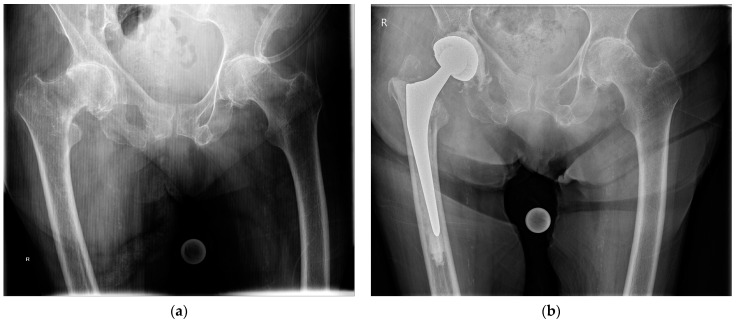
(**a**) Preoperative anteroposterior pelvic radiograph of an intertrochanteric femur fracture with concomitant advanced osteoarthritis of the right hip; (**b**) anteroposterior pelvic radiograph at 1 year after total hip arthroplasty with a cemented femoral stem and a cemented double-mobility acetabular cup.

Concomitant intraarticular pathologies, such as osteoarthritis, inflammatory disease, or femoral head necrosis, are further indications suggested for HA. In these cases, the surgery, on one hand, allows to treat the fracture and the intraarticular condition. On the other hand, the procedure should reduce the stress at the fracture site secondary to stiffening and reduced mobility of the joint, which is believed to be a determinant for nonunion in patients with osteoarthritis [11]. However, the latter indication remains rather unexplored at the moment. Even though osteoarthritis is the most reported concomitant intraarticular disease (Figure 1), no guidance exists about the severity of the symptoms or the degree of osteoarthritis for which treatment of IFFs with HA is recommended.

There is also no consensus about the ideal candidate for HA. The indications reported in the literature are manifold and usually quite broad. Haentjens et al. [12] recommended HA in older patients with a low life expectancy. On the contrary, Zhou et al. [13] reported that HA was contraindicated in patients with a life expectancy of less than two years, severe comorbidities, and an inability to tolerate surgery. In addition, the authors limited the indications to patients >75 years old who were unable to tolerate long-term bed rest. Park et al. [14] had selected patients for HA if they had one or more internal diseases, such as hypertension, cardiovascular diseases, diabetes, and renal diseases. For Xie et al. [15], HA was an option to allow early mobilization and improve the quality of life of patients > 80 years old with preinjury ambulatory capacity who were able to tolerate surgery. Öztürk et al. [16] found a powerful correlation between mortality and patient’s functional status and believed that HA should be reserved for patients with low functional demands who would not tolerate further functional loss in a short time period. However, they suggested that IF should preferably be used in patients with an adequate functional level.

## 3. Surgical Technique

### 3.1. Surgical Approach

There is no guideline as regards the choice of approach for the treatment of IFFs with HA. Surgeon’s experience, preferences, and fracture characteristics are important considerations when selecting the approach. Even though in most studies on the topic, the posterolateral or direct lateral approach is used, other approaches are also viable. For example, Grune et al. [17] used anterior and anterolateral approaches to treat IFFs with total hip arthroplasty (THA).

Each approach has its own pros and cons, particularly regarding the possibilities of fracture and hip joint exposure, the option to extend the approach if needed, and the risk of worsening preexisting damage to the hip abductor muscles. Fichman et al. [18] suggested using a direct lateral approach with a modified trochanter slide osteotomy in cases where the greater trochanter was fractured. This technique allows for excellent exposure by using existing fracture lines and preserving the surrounding musculature. Moreover, an additional osteotomy is rarely needed because frequently, the location of the greater trochanter’s fracture line allows to retract it en bloc.

### 3.2. Implant Selection

There is a wide choice of hip prostheses available on the market, which is also reflected by the heterogeneity of implants reported in the literature for the treatment of IFFs with HA. However, studies comparing different types of devices are scarce. Therefore, it is not possible to provide any conclusive recommendations on the type of hip prosthesis that should be used to treat these fractures. Here, again, the choice of the hip prosthesis is primarily based on the surgeon’s experience, preference, and fracture characteristics but additionally the bone quality and the presence of concomitant intraarticular pathologies.

Among the various types of HA used to treat IFFs, bipolar hemiarthroplasty (BHA) is the most commonly reported. In contrast, THA is rarely used, and its indications and outcomes remain poorly understood. Geiger et al. [19] advised against the use of THA, especially for unstable IFFs, because of a significantly higher rate of dislocation than with BHA. Unfortunately, information about femoral head size used in THA is missing from this publication, which could affect the dislocation rate. Differences in functional outcomes between patients treated with THA or BHA reported by Bonnevialle et al. [20] did not reach statistical significance even when the analysis was restricted to patients with osteoarthritis.

**Figure 2:**
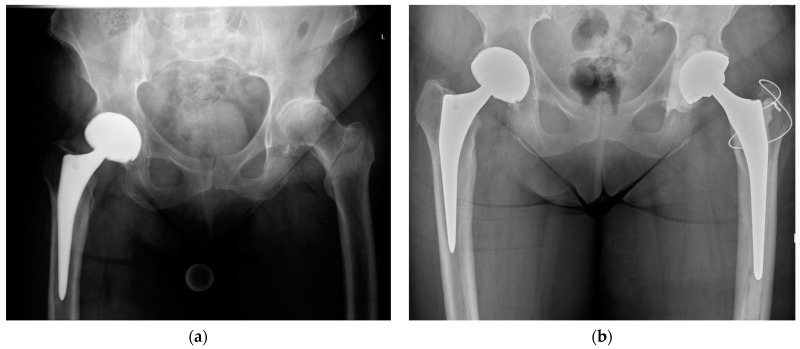
(**a**) Preoperative anteroposterior pelvic radiograph of an intertrochanteric femur fracture with concomitant osteoarthritis of the left hip; (**b**) postoperative anteroposterior pelvic radiograph after total hip arthroplasty with a cemented femoral stem, fixation of the greater trochanter by a figure-of-eight wire cerclage, and a cemented acetabular cup.

Various designs of primary and revision femoral stems have been used successfully for the treatment of IFFs. Grote et al. [21] compared cemented primary stems with revision stems to treat unstable IFFs without finding a significant difference in complication or reoperation rates. Nevertheless, IFFs are often associated with bone loss in the proximal femur that may present a challenge for prosthesis anchoring. Most authors thus recommend adapting the length of the stem to the fracture pattern, particularly to the distal extension of the fracture line and the bone stock (Figure 2). Diaphyseal fixation or calcar replacement stems are suitable options in situations where the metaphysis is significantly compromised. Modular revision femoral stems have the advantage of allowing to first impact the stem into the diaphysis until it is stable so that the length, version, and offset of the overall femoral component can be adjusted through the proximal body in a second step.

Methods of fixation of the femoral stem are also a topic of debate. Bonnevialle et al. [20] noted a better functional outcome of cemented compared to uncemented stems and thus recommended cementing femoral stems in the absence of contraindications. In contrast, Zhou et al. [13] advocated the use of cementless stems to ensure the longevity of the prosthesis over time. In the absence of proven superiority of one method over the other, the choice is up to the surgeon. Both methods of stem fixation have pros and cons.

In older patients with poor bone quality and very thin diaphyseal cortices, cement improves stem fixation by providing immediate stability and additionally decreases the risk of intraoperative periprosthetic fracture. Drawbacks of cement include the risk of fat or bone marrow embolism, of which patients with impaired cardiopulmonary function are particularly at risk; prolonged operative time; and possible delayed fracture healing due to cement in the proximal femur.

Based on improvements in design and materials, cementless implants have gained popularity in recent years. They are often used in hip revision surgery or for periprosthetic fractures. The main advantages of cementless stems are their biological integration, the absence of cement-related complications, and the shorter operating time. However, the risk of intraoperative periprosthetic fracture is higher.

### 3.3. Hip Abductor Mechanism Repair

**Figure 3:**
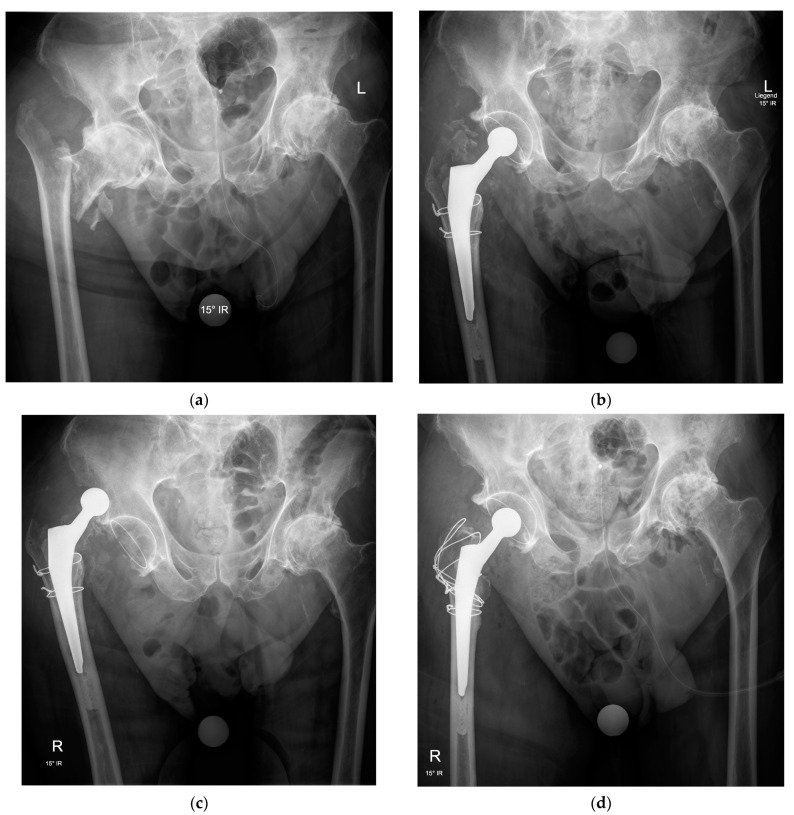
Anteroposterior pelvic radiographs of an intertrochanteric fracture with advanced osteoarthritis (**a**) treated by total hip arthroplasty with a cemented femoral stem, a cemented acetabular cup, and two wire cerclages to stabilize the metaphyseal fragments around the femoral stem but without fixation of the greater trochanter (**b**), resulting in recurrent dislocations in the early postoperative period (**c**) and requiring revision surgery for fixation of the greater trochanter by a figure-of-eight wire cerclage technique allowing restoration of hip stability and healing of the fracture fragments in a good position with no further dislocation (**d**).

Many IFFs leave the greater trochanter as a separate fragment. Considering its importance for proper functioning of the hip joint, appropriate treatment is mandatory. Adequate reduction and fixation of the trochanter fragment improves the stability of the prosthesis and restores the tension in the gluteus medius (Figure 3).

The choice of the fixation technique mainly depends on the surgeon’s habits, fracture characteristics, and bone quality. Several methods are available. Wire or cable cerclage are viable options if fragment size and bone quality are sufficient. A cerclage with non-absorbable sutures is preferred if the fragments are very small, comminuted, or if the bone quality is poor. There are different types of trochanteric plates, but these are generally thick and can cause irritation and pain. Finally, some prosthesis designs include a claw-like attachment to stabilize the greater trochanter [22].

Despite all efforts to repair the fractured greater trochanter, its secondary displacement is a common complication regardless of the repair technique; yet, in older patients with low functional demands, it is well tolerated and often asymptomatic.

## 4. Results

Numerous studies have reported the descriptive outcomes of HA in the treatment of IFFs. However, there is a lack of high-level, evidence-based literature on this subject. Many studies are case series. Other studies compared HA to IF but used mixed or obsolete types of implants, heterogeneous study populations, and no stratification for stable and unstable IFFs. Methodological limitations are also common. The few available randomized trials almost never reported a power analysis [23,24,25], the measurement methods of the different outcome variables were rarely detailed, and the rehabilitation protocol most often differed between HA and IF. In this context, the results reported in the literature are generally inconsistent, and it is difficult to compare studies or to draw clear conclusions for daily practice. In addition, comparative studies specifically evaluating subgroups of patients with unstable IFFs, intraarticular pathologies, or other predictive factors are rare. Yet, this information would be essential to identify patients likely to benefit from one or the other treatment methods.

In the following, we present the outcomes of studies comparing HA to IM, with IM being the IF method of choice for IFFs at higher risk of complications [13,14,18,20,24,25,26,27,28].

Functional outcome is the most frequently studied outcome measure. Surgical treatment of IFFs, whether by HA or IM, leads to improved overall function. This improvement continues for up to two years after surgery but never returns to the preinjury level. Assessments beyond this period have rarely been published. Nevertheless, there are important differences between these two treatment methods concerning the progression of improvement over time [23,29]. HA has a better functional outcome in the early postoperative period. The possibility of immediate full weight-bearing undoubtedly contributes to this result [28]. On the other hand, rehabilitation after IF, especially when an adequate reduction and a stable fixation was not achieved, usually includes a period of protective weight-bearing whose duration varies according to the surgeon and the protocols of each institution. As a result, the positive effect of IM on functional outcome is delayed. However, the increase in function is fast as soon as the patient is able to move without restriction, and the results are similar to HA from 6 to 12 months postoperatively. Beyond this period, outcomes are inconsistent but tend to favor IM, although the reported differences are hardly ever statistically significant. It is assumed that preserving the native hip joint may contribute to this result [30,31].

Complications of surgical treatment of IFFs can be divided into two categories, namely medical and general surgical complications. The rates of medical complications between HA and IM are similar. All organs of the body are potentially affected, with varying severities. Ucpunar et al. [28] found no difference in the rate of postoperative ICU admission between HA and IM. Yet, the authors observed an increase in overall morbidity three months postoperatively in patients treated with HA, those with an American Society of Anesthesiologists (ASA) score 3 or 4, and with a lower level of independence in activities of daily living before the injury. However, this deterioration was temporary and no longer present six months postoperatively. Additionally, it was not reflected in the mortality rates, for which no significant differences were seen at any time point.

IM appears to have higher general surgical complication and reoperation rates than HA. It should be noted that individual studies analyzing these outcomes lack power and that a significant difference emerges when the results are pooled in a meta-analysis [32,33,34]. The type of complications between the two treatment methods is different. The most reported complications of IM are cut-out or protrusion of the lag screw, fixation failure, malunion, and nonunion. The most common complications of HA include dislocation or gross leg length discrepancy. The infection rate is similar for both treatment methods.

Finally, the one-year mortality rate is high regardless of the treatment modality. Results of comparative studies are, however, inconsistent, with some studies reporting a lower mortality rate of IF than HA [33,34] and others a similar mortality rate between both treatment methods [35,36,37]. Female gender, age over 80 years old, lower functional level before the injury, chronic pulmonary diseases, diabetes, ASA score 3 or 4, volume of blood transfusion, increased time between injury and surgery, and total length of hospital stay are other risk factors identified for increased mortality after surgical treatment of IFFs by IM or HA [16,27,38].

## 5. Conclusions

Surgical management by internal fixation remains the mainstay of treatment for the majority of IFFs. HA is an alternative for fractures at high risk of fixation failure after IF or with concomitant intraarticular pathologies. Older patients with low functional demands but ambulatory capacity before the fracture whose condition precludes prolonged bedrest and who are at risk of significant deterioration if their locomotor function cannot be restored rapidly are likely to benefit from HA.

The choice of the surgical technique mainly depends on surgeon’s preferences and fracture characteristics. BHA is the most common type of prosthesis used with primary or revision and cemented or cementless femoral stems. HA has a better early functional outcome and lower rates of surgical complications and reoperations than intramedullary nails. However, the functional outcome and the mortality rate in the longer term tend to favor IM even though the results are inconsistent, and a statistically significant difference cannot always be obtained. The rate of medical complications is similar between the two treatment methods.

Finally, at the moment, there are no guidelines that define the role of HA in the treatment of IFFs in older patients. The current literature only offers an overview of the possibilities of the usage of HA, but methodological limitations are common, and the levels of evidence are low. Further studies are needed to identify, among other things, the IFFs that are at highest risk for fixation failure, the characteristics that determine which patients may benefit most from HA, and the optimal surgical technique.

## Data Availability

Not applicable.
